# Spatial Modeling of PM_10_ and NO_2_ in the Continental United States, 1985–2000

**DOI:** 10.1289/ehp.0900840

**Published:** 2009-06-29

**Authors:** Jaime E. Hart, Jeff D. Yanosky, Robin C. Puett, Louise Ryan, Douglas W. Dockery, Thomas J. Smith, Eric Garshick, Francine Laden

**Affiliations:** 1 Exposure, Epidemiology and Risk Program, Department of Environmental Health, Harvard School of Public Health, Boston, Massachusetts, USA; 2 Channing Laboratory, Department of Medicine, Brigham and Women’s Hospital and Harvard Medical School, Boston, Massachusetts, USA; 3 Department of Epidemiology, Harvard School of Public Health, Harvard School of Public Health, Boston, Massachusetts, USA; 4 South Carolina Cancer Prevention and Control Program, University of South Carolina, Columbia, South Carolina, USA; 5 Department of Environmental Health Sciences and; 6 Department of Epidemiology and Biostatistics, Arnold School of Public Health, University of South Carolina, Columbia, South Carolina, USA; 7 Department of Biostatistics, Harvard School of Public Health, Boston, Massachusetts, USA; 8 Pulmonary and Critical Care Medicine Section, Medical Service, VA Boston Healthcare System, Boston, Massachusetts, USA

**Keywords:** GIS, nitrogen dioxide, outdoor air pollution, particulate matter

## Abstract

**Background:**

Epidemiologic studies of air pollution have demonstrated a link between long-term air pollution exposures and mortality. However, many have been limited to city-specific average pollution measures or spatial or land-use regression exposure models in small geographic areas.

**Objectives:**

Our objective was to develop nationwide models of annual exposure to particulate matter < 10 μm in diameter (PM_10_) and nitrogen dioxide during 1985–2000.

**Methods:**

We used generalized additive models (GAMs) to predict annual levels of the pollutants using smooth spatial surfaces of available monitoring data and geographic information system–derived covariates. Model performance was determined using a cross-validation (CV) procedure with 10% of the data. We also compared the results of these models with a commonly used spatial interpolation, inverse distance weighting.

**Results:**

For PM_10_, distance to road, elevation, proportion of low-intensity residential, high-intensity residential, and industrial, commercial, or transportation land use within 1 km were all statistically significant predictors of measured PM_10_ (model *R*^2^ = 0.49, CV *R*^2^ = 0.55). Distance to road, population density, elevation, land use, and distance to and emissions of the nearest nitrogen oxides–emitting power plant were all statistically significant predictors of measured NO_2_ (model *R*^2^ = 0.88, CV *R*^2^ = 0.90). The GAMs performed better overall than the inverse distance models, with higher CV *R*^2^ and higher precision.

**Conclusions:**

These models provide reasonably accurate and unbiased estimates of annual exposures for PM_10_ and NO_2_. This approach provides the spatial and temporal variability necessary to describe exposure in studies assessing the health effects of chronic air pollution.

Acute exposures to particulate and gaseous air pollutants have been associated with morbidity and mortality in a large number of time-series studies [Pope and Dockery 2006; U.S. Environmental Protection Agency (EPA) 1993, 2004]. There are fewer cohort studies where it has been possible to examine the association of long-term exposures and mortality (Dockery et al. 1993; Finkelstein et al. 2003; Hoek et al. 2002; Jerrett et al. 2005b, 2005c; Laden et al. 2006; Lipfert et al. 2006; Miller et al. 2007; Nafstad et al. 2004; Nyberg et al. 2000; Pope et al. 1995, 2004; Rosenlund et al. 2006). In most long-term studies, exposure assessment has been limited mainly to city-specific average pollution measures or spatial or geographic information system (GIS)–based exposure models in small geographic areas (Adar and Kaufman 2007; Brauer et al. 2003; Briggs et al. 2000; Jerrett et al. 2005a; Liao et al. 2006; Ryan and LeMasters 2007; Su et al. 2008; Wheeler et al. 2008; Wong et al. 2004). One recent study has described a monthly spatiotemporal exposure model for the northeastern United States using a combination of spatial and GIS-derived covariates that outperformed models with spatial smoothing alone (Yanosky et al. 2008, 2009). Another recent report has detailed the use of universal kriging to predict pollution levels for the European Union (Beelen et al. 2009). The purpose of this analysis is to develop nationwide models of annual exposure to particulate matter < 10 μm in diameter (PM_10_) and nitrogen dioxide, using a combination of spatial smoothing and regression of GIS-derived covariates. To date, few countrywide models have been available for these pollutants over our time scale of interest (1985–2000). We apply the model to the addresses of the workers in the Trucking Industry Particle Study (Garshick et al. 2008; Laden et al. 2007), a retrospective cohort study of male U.S. unionized trucking company workers, to illustrate its potential use in exposure assessment for long-term epidemiologic studies with members spread over the continental United States.

## Methods

### The Trucking Industry Particle Study

Details of the Trucking Industry Particle Study (TrIPS) are provided elsewhere (Garshick et al. 2008; Laden et al. 2007). Briefly, using personnel records from four large companies we identified 54,973 males with at least 1 day of work in 1985. Information was available on demographic variables, daily job and work location, and residential home address. Using an outside vendor (TeleAtlas, Lebanon, NH), we geocoded the last known residential addresses of 53,822 members living within the continental United States to at least the ZIP code level.

### Pollutant data

We obtained information on annual average PM_10_ (parameter codes 81102 and 85101) and NO_2_ from the U.S. EPA Air Quality System (AQS). The U.S. EPA provided these annual averages on a set of DVDs compiled in 2004 for U.S. EPA Science to Achieve Results program grant 83054501-0. Data from 1985–2000 were used for this study if an annual mean was reported, regardless of the primary monitoring objective of the monitor. All monitors in the continental United States were included, because excluding monitors such as those located near point or mobile sources would prevent us from incorporating all sources of spatial variability represented in the monitoring network. Latitude and longitude of each monitor were obtained from the AQS database and used to map the monitor locations using ArcGIS (version 9.2; ESRI, Redlands, CA). All monitors were checked for latitude/longitude accuracy and precision to the county level before inclusion.

### Modeling approach

We used generalized additive models (GAMs) to predict annual outdoor levels of PM_10_ and NO_2_ using smooth spatial surfaces and GIS-derived covariates. GAMs use semiparametric methods to model nonlinear, one-dimensional, and multidimensional functions using penalized splines (Hastie and Tibshirani 1990; Wood 2003, 2004, 2006). For both pollutants, models were constructed using 90% of the available monitoring locations for each calendar year. The remaining randomly selected 10% of monitors were used to perform cross-validation as described below.

First, the average spatial surface for each pollutant, 1985–2000, was generated in a GAM containing a bivariate thin-plate spline of the projected *x*- and *y*-coordinates of the monitoring locations and indicator variables for calendar year to adjust for temporal trends (Wood 2006). To obtain information on fine-scale long-term spatial patterns, we included one-dimensional penalized splines for *a priori* selected GIS-derived time-invariant covariates. The covariates we considered included distance to road, population density, elevation, surrounding land use, distance to and emission from power plants, and variables for census region of the country (northeast, west, south, and midwest) to adjust for regional patterns. These variables have previously been shown to be important predictors of ambient pollution (Adar and Kaufman 2007; Jerrett et al. 2005a; Ryan and LeMasters 2007; Yanosky et al. 2008, 2009). Each characteristic was assigned to the monitoring locations using ArcGIS.

Information from the StreetMap data set (ESRI) was used to determine distance to the nearest road. Road segments were first classified by U.S. Census Feature Class Code as A1 (primary roads, typically interstates, with limited access), A2 (primary major, noninterstate roads), or A3 (smaller, secondary roads, usually with more than two lanes) (U.S. Census Bureau 1993). The distance from each location to the nearest road of each road class was then calculated in meters. Land use data were compiled from the U.S. Geological Survey (USGS) 1992 National Land Cover Dataset (USGS 2007b), which provides data on 19 categories of land use in raster image files with 1 arc-sec (about 30 m) spatial resolution (Vogelmann et al. 2001). The proportion of low-intensity residential, high-intensity residential, and industrial/commercial/transportation land uses within 1 km of each location was calculated. Population density values were assigned to each monitoring location using data from the 2000 U.S. Census at the block group level (U.S. Census Bureau 1993). Elevation data for each location were compiled from the USGS National Elevation Dataset (USGS 2007a). Information on the tons of nitrogen oxides emitted annually from all U.S. power plants in 2004 was obtained from the U.S. EPA 2006 Emissions and Generation Resource Integrated Database (U.S. EPA 2007a). The distance to and the emissions from the nearest facility were determined for each NO_2_ monitoring location.

Each potential covariate (or groups of covariates for distance to road, land use, and power plant distance/emissions) was first considered separately in models that included the bivariate spline for the 1985–2000 spatial surface and the indicator variables for calendar year. We constructed multivariate models including all covariates that were statistically significant (*p* < 0.05) and led to a higher adjusted model *R*^2^. If covariates were no longer significant when included in the multivariate model, we omitted them unless they led to better model fit as determined by Akaike’s information criterion (AIC) and cross-validation testing.

To assess annual differences from the long-term spatial patterns of pollution, we first calculated the residuals from the final long-term multivariate GAM models. Then, for each calendar year, we created a bivariate smooth of the residuals using a two-dimensional thin-plate spline. Therefore, the annual average pollution at any location was predicted using the sum of the prediction from the long-term average surface/GIS-derived covariates and the prediction from the calendar-year specific residual spatial variability surface.

To perform cross-validation, we used regression parameters from the final models and the annual spatial surfaces to predict annual pollutant levels at the 10% of monitoring locations that were held out from the original models. We assessed the potential bias of each final model by calculating the prediction error as the difference between the observed and predicted values at each cross-validation monitoring location. We also assessed bias in the models by examining the intercept and slopes from linear regression of the predicted values on the measured values. The precision of the model was estimated by taking the square root of the mean of the squared prediction errors (RMSPE). In addition, a cross-validation *R*^2^ was obtained using the squared Pearson correlation between the measured values at the held-out observations and the model predictions.

For comparison, we also predicted exposures using a simpler spatial interpolation method, inverse distance weighting (IDW), which had been frequently used in the air pollution literature. For the IDW models, the annual predictions for any given location (cross-validation monitor location or cohort member address) were calculated by taking the average of the measured value at each monitor location times the inverse of the squared distance between each location and each monitor. IDW modeling was performed in ArcGIS (Johnston et al. 2004). The bias and precision of this simpler exposure modeling method was determined using cross-validation.

After the final GAM models were determined and cross-validated, the regression parameters were used to predict annual pollutant levels at the 53,822 residential addresses of the TrIPS cohort members. For comparison, IDW was also used to predict annual pollutant levels at the residential addresses. Statistical analyses were performed in PC SAS version 9.1 (SAS Institute Inc. 2006) and Unix R 2.7.0 (R Development Core Team 2006).

## Results

**Figure 1:**
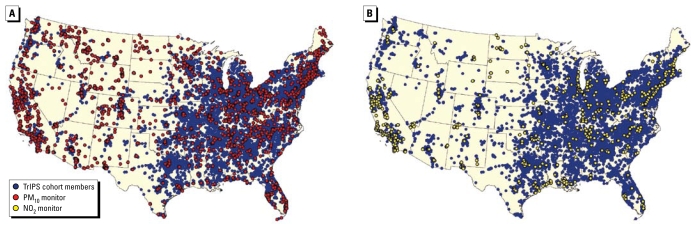
TrIPS cohort members and monitoring locations for PM_10_ and NO_2_.

The number of monitors used in the models and annual distributions of pollutant levels are shown in Table 1. The levels of both pollutants decreased over time. The median value of PM_10_ in 1985 was 38.2 μg/m^3^, and it fell to 23.0 μg/m^3^ by 2000 (a 40% decrease). The median NO_2_ level decreased 23% over the same period, from 19.0 ppb to 14.6 ppb. The distributions of the GIS-derived covariates at the monitor locations considered in the GAM models are shown in Table 2. The covariate distributions were quite similar for both sets of monitors. As shown in Figure 1, the cohort participants are located throughout the continental U.S., and most live close to the monitoring locations. Specifically, the cohort members lived a median distance of 10.2 km from PM_10_ monitoring sites and 16.6 km from NO_2_ sites. Seventy-five percent of the cohort was no more than 21.1 km from a PM_10_ monitor included in the model and 35.6 km from an NO_2_ monitor included in the model.

### PM_10_

The model with only the spatial spline and calendar year indicator variables had a model *R*^2^ of 0.48. Region of the country, distance to all three census classes of road, block group population density, elevation, proportion of low-intensity residential, high-intensity residential, and industrial, commercial, or transportation land use within 1 km were all statistically significant independent predictors of measured PM_10_ concentrations in univariate models. In a multivariate model, all predictors except population density (*p* = 0.15) remained statistically significant predictors of measured PM_10_ annual concentrations (Table 3). Population density was removed from the final model, because it did not increase the cross- validation *R*^2^ or model fit as determined by AIC. The final model had an *R*^2^ of 0.49. Increases in the proportion of surrounding land use used for high-intensity residential or for industrial, commercial, or transportation uses were associated with increases in measured PM_10_ levels. Increases in all other covariates were associated with decreases in measured PM_10_. The cross-validation *R*2 of the final model was 0.55. The median [and interquartile range (IQR)] prediction error of the final model was 0.24 (7.0) μg/m^3^. The intercept and slope from the regression of observed and predicted measurements were 1.49 and 0.94, respectively, and the RMSPE was 9.1 μg/m^3^. A plot of the observed versus expected values from the cross-validation is presented in Supplemental Material, available online (doi:10.1289/ehp.0900840.S1 via http://dx.doi.org/).

### NO_2_

The model with only the spatial spline and calendar year indicators had a model *R*^2^ of 0.73. Region of the country, distance to road, block group population density, elevation, surrounding land use, distance to nearest NO_x_-emitting power plant, and the level of emissions from that power plant were all statistically significant predictors of measured NO_2_ concentrations in univariate models. In a multivariate model, all predictors remained statistically significant predictors of measured NO_2_ annual concentrations (Table 3). The final multivariate model had an *R*^2^ of 0.88. Increases in the block group population density, NO_x_ emissions of the nearest power plant, and the proportion of surrounding land use used for low- or high-intensity residential or for industrial, commercial, or transportation uses were associated with increases in measured NO_2_ levels. Increases in all other covariates were associated with decreases in measured NO_2_. The cross validation *R*^2^ of the final model was 0.90. The median (and IQR) prediction error of the final model was 0.10 (3.7) ppb, the intercept and slope of the regression of observed and predicted measurements were 0.00 and 1.04, and the RMSPE was 3.5 ppb. A plot of the observed versus expected values from the cross-validation is presented in Supplemental Material (doi:10.1289/ehp.0900840.S1).

### Comparison with IDW

A summary of the cross-validation parameters for the IDW exposure models is presented in Table 4. For both pollutants, the cross-validation *R*^2^ of the IDW model (*R*^2^ = 0.44 for PM_10_ and 0.67 for NO_2_) was lower than those from the GAMs (*R*^2^ = 0.55 for PM_10_ and 0.90 for NO_2_). For PM_10_, the slope from regression for the IDW model was 0.76 and the slope for the GAM was 0.94, indicating greater accuracy. The median prediction error for the IDW model was almost half that of the GAM, also indicating greater accuracy, but the RMSPE was higher, indicating lower precision. In contrast, for NO_2_ the IDW prediction error was 10-fold higher than the GAM, and the RMSPE was almost twice as large.

### TrIPS cohort exposures

The distribution of the GIS-derived variables for the residential addresses (*n* = 53,822) of the TrIPS cohort is presented in Table 5. The home addresses tended to be further away, on average, from each of the census road classes and from power plants than the monitors used to develop the models. The addresses were also located in areas with a lower proportion of high-intensity residential or industrial, commercial, or transportation land use, and the addresses were located further away from power plants than monitors, with lower annual emissions of NO_x_ from the nearest plant, on average. The distributions of the covariates tended to be tighter than those of the monitoring locations but were not significantly different.

**Figure 2:**
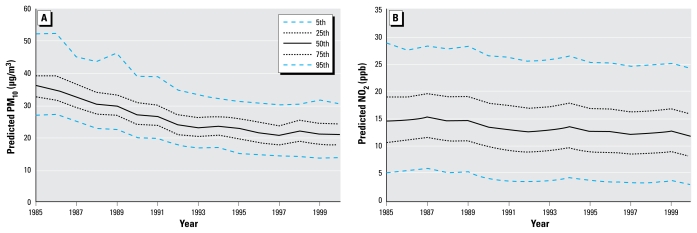
Distrubution of annual GAM-predicted PM_10_ (*A*) and NO_2_ (*B*) values (by percentile) at the TrIPS cohort addresses.

**Figure 3:**
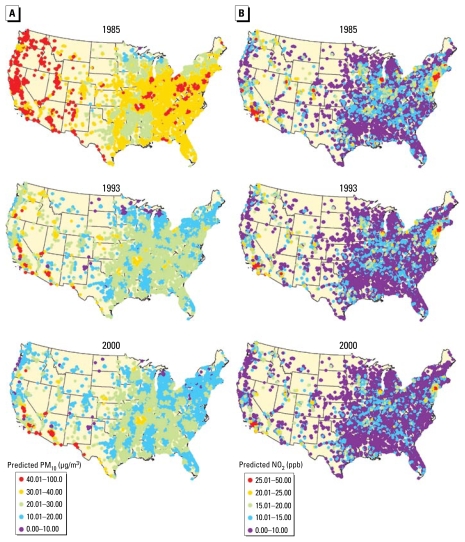
Annual GAM-predicted PM_10_ (*A*) and NO_2_ (*B*) values at the TrIPS cohort addresses at the beginning (1985), middle (1993), and end (2000) of follow-up.

**Figure 4:**
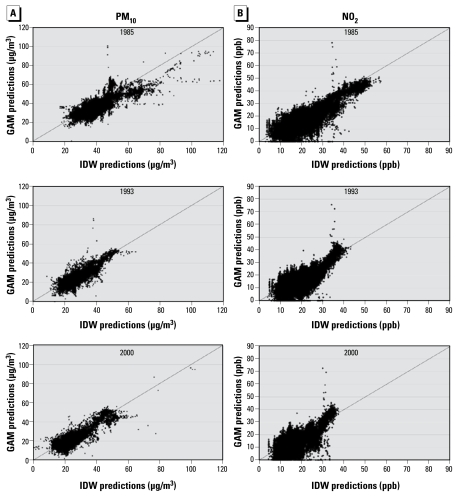
Comparisons of the distribution of GAM- and IDW-predicted values for PM_10_ (*A*) and NO_2_ (*B*) at TrIPS cohort residential addresses at the beginning (1985), middle (1993), and end (2000) of follow-up.

Figure 2 shows the distribution of the pollution values for each year at the cohort addresses. The mean predicted levels of the two pollutants decreased over the follow-up period, although there was little change in the overall spread of the distributions. The spatial distributions of the predictions for both PM_10_ and NO_2_ are shown in Figure 3. At all three time points shown, PM_10_ values are higher in the western half of the United States than in the east. For NO_2_, however, the levels in all time periods are highest in major cities. To compare the two prediction methods, Figure 4 shows the cohort predictions for PM_10_ at base-line (1985), midpoint (1993), and last year of follow-up (2000). There is moderate correlation between the results of the GAM and IDW PM_10_ models, although the IDW models tend to be lower than the predictions of the GAMs (thus their lower slope of 0.76 vs. 0.94 for the GAM when both are compared with measured concentrations). The Spearman correlations between the two prediction types were 0.66 for 1985, 0.64 for 1993, and 0.77 for 2000. As shown in Figure 4, there is also moderate correlation between the GAM and IDW NO_2_ models. Specifically, the Spearman correlation is 0.63 for 1985, 0.53 for 1993, and 0.51 for 2000. Overall, the IDW models tend to be lower than the GAM predictions and tend to have less variance (heterogeneity).

## Discussion

Our results show that GAMs with a combination of spatial smoothing and GIS-derived covariates are a practical method for predicting annual outdoor air pollution values for a cohort dispersed across the continental United States. The PM_10_ and NO_2_ GAM models were reasonably accurate and precise. The final model for NO_2_ had a model *R*^2^ of 0.88 and a cross-validation *R*^2^ of 0.90, whereas the final model *R*^2^ for PM_10_ was 0.49 and the cross-validation *R*^2^ was 0.55. Overall, the GAMs for both PM_10_ and NO_2_ outperformed the simpler IDW models, although there was a greater difference in the performance of the two modeling approaches for NO_2_.

As expected, based on the growing literature of land-use regression models, many GIS-derived predictors were important in the pollution models. Distance to the nearest road of each road class, distance to and emissions from the nearest power plant, and land-use terms defining the surrounding area, variables previously shown to represent major sources of ambient NO_2_ in the United States (U.S. EPA 2007b), were all statistically significant predictors of NO_2_. In PM_10_ models, distance to the nearest road of each road class was the most important class of predictors, likely representing traffic, an important local source of particulate matter (U.S. EPA 2004). These covariates did not improve the model *R*^2^ as much for PM_10_ as they did for NO_2_. It is possible that there are other important sources of PM_10_ that we have not included (e.g., sea salt, crustal materials) that would improve the model *R*^2^ more.

A growing number of studies have used spatial smoothing methods or models based on GIS-derived variables to predict ambient air pollution levels for use in epidemiologic studies (Adar and Kaufman 2007; Jerrett et al. 2005a; Ryan and LeMasters 2007). Many of these studies have relied on proximity to specific pollution sources or monitoring locations to assign exposures. Others have focused on characterizing pollution from a specific source, typically on-road vehicles (Hoek et al. 2001). The most commonly used GIS-based methods have used information on traffic volume and distance to roadways as surrogates of exposure (Adar and Kaufman 2007; Bayer-Oglesby et al. 2006; Forastiere and Galassi 2005; Garshick et al. 2003; Kan et al. 2007; Nitta et al. 1993; Oosterlee et al. 1996; Venn et al. 2005). In many of these studies, distance to road is divided into categories, or individuals are classified as exposed or not exposed, based on an *a priori* chosen distance. This method likely leads to exposure misclassification in many of these studies and is likely also quite sensitive to the buffer or category size selected. Another popular GIS-based exposure method is land use regression (Briggs et al. 1997; Hoek et al. 2001; Ryan and LeMasters 2007; Ryan et al. 2007; Su et al. 2008). This approach is typically used in smaller areas to model local spatial variability, and roadway networks and traffic are often inputs to these models, although some also include information on surrounding land use, meteorology, and ambient air pollution monitoring locations. Other studies have used spatial smoothing techniques of the ambient measurements in single cities or counties (Jerrett et al. 2005b; Meng et al. 2007). Although direct comparisons are not appropriate, our NO_2_ model *R*^2^ of 0.88 is higher than those observed in many land-use regression models (0.52–0.76) (Briggs et al. 2000; Cyrys et al. 2005; Gilbert et al. 2005; Rosenlund et al. 2008) or in an EU-wide model based on ordinary kriging (Beelen et al. 2009).

On a larger spatial scale, in an exposure assessment for the Women’s Health Initiative, kriging in ArcGIS was used to generate daily PM_2.5_ and PM_10_ estimates for the entire continental United States for the year 2000 (Liao et al. 2006; Szpiro et al. 2007). For PM_10_, the authors report a median prediction error of 0.04 μg/m^3^ and an RMSPE of 19.48 μg/m^3^. In a recent exposure assessment for the Nurses’ Health Study, a combination of spatial smoothing and GIS-derived covariates was used to produce monthly predictions of PM_10_ 1988–2002 for residences in the northeastern United States (Yanosky et al. 2008). This model has a mean prediction error of −0.4 μg/m^3^ and an RMSPE of 6.4 μg/m^3^ across the entire region, with no discernable differences by state or level of urbanization. Our models are similar to this modeling approach: Both include spatial smoothing and GIS-based covariates to generate predictions. The Yanosky model allows the generation of monthly estimates of PM_10_ through a complex spatiotemporal model and allows the inclusion of time-varying covariates and control for seasonality. In contrast, although the model presented here also uses spatial smoothing and GIS-based covariates, it is more appropriate for annual means and is less computationally intensive. Therefore, for PM_10_, the amount of bias [measured by average (mean or median) prediction error] and precision (measured by RMSPE) in our final model are comparable to that of other studies in the United States.

Our exposure model has several important limitations. We rely on air pollution data from existing networks that are not uniformly distributed across the continental United States. However, the measures of precision and accuracy determined by cross-validation for the held-out monitoring locations indicated good predictive performance of the models. Additionally, most of the members of the specific cohort we are using in this analysis live close to monitoring locations, so the mismatch between monitor and subject locations is unlikely to be a large source of error in exposure for our chosen application. For studies where the cohort is located much further from monitoring locations, this would likely be a larger source of error. In focusing our modeling on annual means, we are likely missing important seasonal and temporal variability occurring within each year. In years with fewer monitoring locations, it is possible that our model is underpowered to detect annual differences from the long-term spatial trends; however, in later years, only 20–40 degrees of freedom were needed to fit these surfaces, so this may not be a large issue. Our model also does not include information on time-varying covariates (such as point-source pollution or weather, especially wind direction and speed, mixing height, and precipitation) or interactions between our chosen covariates and calendar year. It is likely that information on these factors would improve the predictive ability of our model; however, it would require a different modeling approach than the one we have chosen. By treating population density, distance from road, and land use as time invariant, we are assuming that these did not vary during the study period. This is not likely to be true and will lead to increased error in areas with rapidly changing infrastructure during this time period. Finally, we are using a spatial smoothing model for the entire continental United States. It has been suggested that regional models may be more appropriate for the continental United States (Szpiro et al. 2007); however, it has been shown that for daily predictions, regional models do not substantially outperform a single countrywide model (Liao et al. 2006). Our models are adjusted for region of the country (using indicator variables), and although including region did improve the fit of the models, the regional terms themselves were not significant.

## Conclusions

In conclusion, our air pollution exposure model combining spatial smoothing techniques and GIS-based predictors is a useful way to provide estimates of U.S.-wide annual exposures for PM_10_ and NO_2_. These models can be used to produce reasonably accurate and precise measures of pollution at the residential addresses of participants in epidemiologic studies focusing on the adverse effects of constituents of air pollution as far back as 1985.

## Figures and Tables

**Table 1:** Number and percentile distribution of measured annual mean values for all PM_10_ and NO_2_ monitors included in the generalized additive and inverse distance weighted models.

	PM_10_ (μg/m^3^)						NO_2_ (ppb)					
Year	No.	5th	25th	50th	75th	95th	No.	5th	25th	50th	75th	95th
1985	369	18.0	30.1	38.2	46.9	84.4	320	3.3	11.1	19.0	24.9	39.2
1986	567	20.6	30.2	37.5	45.6	72.9	310	3.3	10.1	18.3	24.6	35.2
1987	881	19.2	28.1	34.8	42.1	61.5	268	3.4	11.9	19.3	26.2	39.3
1988	996	17.1	25.8	32.0	39.7	56.7	298	3.0	10.1	18.9	26.1	39.1
1989	1,127	15.0	25.8	30.9	37.5	57.1	308	3.1	12.0	19.6	26.2	38.9
1990	1,319	13.7	22.3	27.3	34.1	48.3	326	3.7	10.1	17.4	23.4	35.4
1991	1,379	13.3	22.9	27.8	33.7	47.9	325	3.3	9.8	16.2	23.8	34.5
1992	1,509	12.2	20.9	25.3	31.0	43.5	339	3.4	10.1	16.3	22.8	35.0
1993	1,513	11.6	20.1	24.9	29.6	42.1	357	3.7	9.1	15.9	22.2	33.9
1994	1,595	12.4	20.2	24.7	30.0	42.4	363	3.7	9.4	16.4	23.5	34.7
1995	1,641	11.3	18.9	23.6	29.1	42.5	373	3.8	9.5	16.0	21.8	33.0
1996	1,659	12.1	19.1	23.3	27.9	41.4	380	3.8	9.2	15.6	21.5	33.5
1997	1,737	11.0	18.9	22.8	27.6	43.2	385	4.0	9.2	14.7	20.0	32.4
1998	2,722	11.8	19.4	23.5	28.3	41.8	400	3.7	8.9	14.5	20.4	32.5
1999	2,419	11.4	18.9	23.7	29.0	50.6	400	3.8	9.5	15.8	21.8	32.5
2000	2,133	11.3	18.5	23.0	28.5	48.2	392	3.6	9.2	14.6	20.2	30.4
ALL	23,565	12.3	20.4	25.3	31.9	48.9	5,544	3.5	9.7	16.5	23.0	34.9

**Table 2:** Summary of the GIS-derived covariates for the PM_10_ and NO_2_ monitors evaluated in exposure models, by percentile.

	PM_10_					NO_2_				
Covariate	5th	25th	50th	75th	95th	5th	25th	50th	75th	95th
Block group population density (people/km^2^)	2	203	1,643	3,917	9,343	5	132	2,139	4,919	14,790
Elevation (meters above sea level)	4	84	214	684	1,836	4	28	150	275	1,314
Land use within 1 km (%)										
Low-intensity residential	0.0	4.6	19.2	36.9	61.9	0.0	2.5	17.6	35.0	63.3
High-intensity residential	0.0	0.0	3.4	17.7	45.0	0.0	0.0	3.3	18.0	47.6
Industrial, commercial, transportation	0.0	5.0	15.8	30.9	59.1	0.0	3.2	12.5	26.0	57.4
Distance to nearest road (km)										
A1 road	0.13	0.8	2.5	11.4	75.6	0.16	0.7	2.0	5.3	43.0
A2 road	0.06	0.4	1.7	6.4	34.9	0.09	0.7	2.6	7.2	30.6
A3 road	0.04	0.4	1.2	3.0	13.2	0.05	0.4	1.4	3.5	13.1
Distance to nearest power plant (km)						1.42	3.7	8.39	17. 7	39.8
NO_x_ emissions of nearest power plant (tons)						0.9	24.1	113.6	893.8	12275.4

**Table 3:** Summary of the fit and statistical significance of the GIS-derived variables included in the final generalized additive models.^a^

	PM_10_ model (μg/m^3^)	*p*-Value^b^	NO_2_ model (ppb)	*p*-Value^b^
Model *R*^2^	0.49		0.88	
Cross-validation *R*^2^	0.55		0.90	
Regression intercept and slope^c^	1.49, 0.94		0.00, 1.04	
Median (IQR) prediction error	0.24 (7.0)		0.10 (3.7)	
RMSPE	9.1		3.5	
Final model GIS-derived variables	Direction of association^d^		Direction of association d	
Population density			Positive^d^	< 2 × 10^−16^
Elevation	Negative^d^	< 2 × 10^−16^	Negative	< 2 × 10^−16^
Percent low-intensity residential land use within 1 km	Negative	2.04 × 10^−13^	Positive	1.79 × 10^−6^
Percent high-intensity residential land use within 1 km	Positive	1.26 × 10^−5^	Positive	< 2 × 10^−16^
Percent ICT land use within 1 km	Positive	< 2 × 10^−16^	Positive	< 2 × 10^−16^
Distance to A1 road	Negative	0.05	Negative	< 2 × 10^−16^
Distance to A2 road	Negative	5.03 × 10^−8^	Negative	5.01 × 10^−16^
Distance to A3 road	Negative	4.47 × 10^−3^	Negative	4.50 × 10^−3^
Distance to power plant^e^			Negative	1.66 × 10^−9^
NO_x_ emissions from nearest plant			Positive	9.77 × 10^−4^

Abbreviations: ICT, percentage of land used for industrial, commercial, or transportation; IQR, interquartile range; difference between the 75th and 25th percentile. Population density excluded from final PM_10_ model.

a All models also include indicator variables for region of the country.

b *R* does not provide exact *p*-values for those < 2 × 10^−16^.

c Regression slope is linear regression of observed measurements at the hold-out locations on model predictions at those locations.

d Negative or positive.

e Distance to and NO_x_ from the nearest power plant were not considered for PM_10_.

**Table 4:** Comparison of the predictive performance of general additive generalized additive models (GAM) and inverse distance weighted exposure models.

	PM_10_ (μg/m^3^)		NO_2_ (ppb)	
Exposure model	GAM	IDW	GAM	IDW
Cross-validation *R*^2^	0.55	0.44	0.90	0.67
Regression intercept and slope^a^	1.49, 0.94	6.44, 0.76	0.00, 1.04	0.00, 1.00
Median (IQR) prediction error	0.24 (7.0)	0.11 (6.1)	0.10 (3.7)	1.00 (7.5)
RMSPE	9.1	10.5	3.5	6.5

a Regression slope is linear regression of observed measurements at the hold-out locations on model predictions at those locations.

**Table 5:** Summary of GIS-derived covariates, by percentile, for TrIPS cohort member residential addresses.

	Covariate distribution				
Covariate	5th	25th	50th	75th	95th
Block group population density (people/km^2^)	42	300	1,686	4,382	10,162
Elevation (m above sea level)	14	125	209	294	1,126
Land use within 1 km (%)					
Low intensity residential	0.0	6.2	23.7	41.8	66.5
High intensity residential	0.0	0.0	3.2	15.0	48.2
Industrial, commercial, transportation	0.0	1.0	4.6	11.7	28.3
Distance to nearest road (km)					
A1 road	0.3	1.3	3.1	7.1	23.5
A2 road	0.2	1.0	2.8	7.1	17.6
A3 road	0.1	0.6	1.7	6.3	8.1
Distance to nearest power plant (km)	2.8	6.8	11.7	19.4	39.9
NO_x_ emissions of nearest power plant (tons)	0.3	7.9	76.0	720.5	8,934.5
